# Potentially Inappropriate Use of Transdermal Fentanyl in Working-Age and Older Adult Populations with Non-Cancer Pain: Nationwide Cross-Sectional Study

**DOI:** 10.2196/63960

**Published:** 2025-05-28

**Authors:** Seunghyun Cheon, Seo-Yong Choi, Kyu Ri Kim, Han-Gon Choi, Jee-Eun Chung

**Affiliations:** 1 College of Pharmacy and Institute of Pharmaceutical Science and Technology, Hanyang University, 55 Hanyangdahak-ro, Ansan, 15588, Republic of Korea, 82 314005816, 82 314005958

**Keywords:** transdermal fentanyl, prescription opioid, potentially inappropriate use, claim data, working age, older adult, Lorenz curves, Gini coefficients

## Abstract

**Background:**

As a potent opioid analgesic, fentanyl transdermal patches (FTDs) have been widely used in patients with moderate to severe pain. However, increasing concerns about the opioid epidemic have made it important to strengthen the rational use and management of FTDs.

**Objective:**

Our objective was to investigate the trends in the use of FTD and to evaluate potentially inappropriate FTD use in patients with noncancer pain, based on established evaluation criteria, referring various recommendations and guidelines.

**Methods:**

A nationwide cross-sectional study was conducted using a national insurance claims database from 2014 to 2020. The study included patients who were prescribed FTDs at least once a year in an outpatient setting, while excluding cancer or pediatric patients. To identify potentially inappropriate use, we developed evaluation criteria based on the established recommendations for the safe use of prescription opioids in patients with noncancer pain and assessed each patient’s compliance. The working-age and older adult groups were compared to evaluate the differences in FTD use, and modified Lorenz curves and Gini coefficients were used to assess the equality of FTD use.

**Results:**

A total of 5386 patients and their 19,800 reimbursements were included in the study. The number of patients with FTDs decreased from 58.6 to 53.7 per 100,000 registrants between 2014 and 2020. Meanwhile, the number of reimbursements increased by 7.4%, from 203.2 to 218.1 per 100,000 registrants during the same period. The working-age patients had an average of 3.9 reimbursements per year, with an average morphine milligram equivalent per day (MME/day) of 64.4 for each reimbursement. The older adult patients had an average of 3.5 reimbursements per year, and their average MME/day was 47.9. As a result of applying the evaluation criteria, 567 (24.5%) working-age patients and 531 (17.3%) older adult patients were identified as potentially inappropriate FTD users. Among patients with multiple FTD prescriptions, the working-age group with potentially inappropriate FTD use had significantly higher estimated MME/day than the older adult group (*P*<.001). The modified Lorenz curve showed that more than 70% of the total FTDs used in the working-age group were consumed by those with potentially inappropriate FTD use, while older adult group with potentially inappropriate FTD use accounted for less than 50% of the total older adult patients. The working-age patients also had a higher Gini coefficient than the older adult group, indicating unequal use of FTDs (0.461 vs 0.406).

**Conclusions:**

Although the number of patients receiving FTD prescriptions has decreased during the study period, the total amount of FTDs consumed increased, suggesting that caution is warranted. This study also highlights the potential for inappropriate FTD use in working-age patients. Further research is needed to quantify and qualify the risk factors in patients with potentially inappropriate use, given the clinical rationale associated with prescribing FTDs.

## Introduction

### Background

In recent years, the opioid crisis has emerged as a significant global health challenge [1,2], with an increasing number of patients with opioid use disorders (OUDs) leading to substantial socioeconomic burdens. While the exact cause of the opioid crisis remains controversial, the US government attributes it primarily to lenient management and chronic use of prescription opioids [3-5]. Wei et al [6] reported that approximately 65% of adult patients under 65 years diagnosed with OUD or overdose in the United States had used prescription opioids. Several studies have also examined the patterns of prescription opioid use in different countries, highlighting concerns about excessive consumption and the potential for opioid crises in several regions [7-10]. An analysis of trends in global prescription opioid consumption using pharmaceutical sales data found excessively high use in some countries [11], while another study using VigiBase raised concerns about the possibility of an opioid crisis in several countries [12], and Ju et al’s [13] study documented a 4.0% annual increase in global prescription opioid usage.

Similarly, the use of prescription opioids has steadily increased in South Korea [7,13,14]. The implementation of the Narcotics Information Management System (NIMS) since 2018 has aimed to monitor the entire process from production to distribution, prescription, and dispensing of scheduled drugs, including prescription opioids [15]. However, approximately 15% of noncancer patients receiving prescription opioids in South Korea were evaluated to have been prescribed inappropriately [16]. This percentage is comparable to the 18% of Medicaid beneficiaries in the United States who were found to have received potentially inappropriate opioid prescriptions before the declaration of the opioid crisis [17]. In addition, the incidence of opioid abuse in noncancer patients with chronic prescription opioid use was reported to be 19.9 per 100 person-years [18]. The illegal use of FTDs among adolescents has emerged as a social concern [19]. In response to these problems, the Korea Ministry of Food and Drug Safety established the recommendations for safe use of prescription opioids for noncancer pain, based on recommendations from various countries such as the Centers for Disease Control and Prevention (CDC) guidelines [20].

Fentanyl and its analogues have been particularly highlighted in the recent opioid epidemic due to their potency and potential for abuse. Despite reductions in the dispensing of other prescription opioids in the United States, fentanyl prescriptions have shown minimal decline or even increases in certain health care settings [21]. In Australia, a significant percentage (4%) of deaths attributed to prescription opioids were linked to fentanyl alone, with a significant portion of fentanyl-related deaths associated with prescribed fentanyl [22,23]. Despite the high risk of abuse, the fentanyl prescriptions have increased rapidly, especially in high-income countries [11,16], and research on the appropriateness of FTD use in the real world remains limited. The most commonly prescribed form of fentanyl is the fentanyl transdermal patch (FTD). FTDs are most widely used to control chronic pain because of their extended-release property that steadily releases the drug over 72 hours to retain a high plasma concentration [24]. Therefore, it is necessary to investigate whether the use and management of FTDs for medical purposes has been appropriate. Given the differences in health profiles, such as the prevalence of chronic pain and prescription opioid use, between older adults and working-age adults [25,26], it is essential to analyze FTD use in these 2 groups separately. Furthermore, opioid abuse and its socioeconomic impacts among working-age adults have become increasingly problematic in recent years [27,28]. Comparing these 2 groups might provide a more precise understanding of FTD use patterns.

### Purpose

We aimed to investigate the trend of FTD usage and evaluate the potentially inappropriate FTD use in patients with noncancer pain based on established evaluation criteria by referring to various recommendations and guidelines.

## Methods

### Study Design

We conducted a nationwide cross-sectional study using the national health insurance claim database. This study was conducted in accordance with the Strengthening the Reporting of Observational Studies in Epidemiology (STROBE) guidelines for cross-sectional studies (Multimedia Appendix 1).

### Data Sources

We used the Health Insurance Review and Assessment Service-National Patient Sample (HIRA-NPS) claim database from 2014 to 2020. The HIRA-NPS claim database is derived from the national health insurance system, which covers approximately 98% of the South Korean population [29]. It is constructed through stratified random sampling based on sex and age group from all individuals who enrolled in the Korean national health insurance by the HIRA and provided to researchers. The HIRA-NPS datasets contain information on approximately 1,400,000 registrants per year for 2014‐2018 (sampling ratio, 3%) and 1,000,000 registrants per year for 2019‐2020 (sampling ratio, 2%). The information of registrants in the HIRA-NPS includes an encrypted personal identification code, reimbursement codes, sex, age, diagnoses, prescription information, and health care institution information [30].

### Study Patients

Patients who had been prescribed FTDs at least once a year in an outpatient setting were included. To investigate FTD use for noncancer pain only, the participants with a history of cancer diagnosis were excluded from each year of data. Pediatric patients younger than 20 years were also excluded. The patients were classified into working-age (<65 years of age) and older adult (≥65 years of age) groups. Subsequently, each group was divided into a single-prescription group and a multiple-prescription group: working-age patients with a single FTD prescription group (WS), working-age patients with multiple FTD prescriptions group (WM), older adult patients with a single FTD prescription group (OS), and older adult patients with multiple FTD prescription group (OM). It was evaluated whether the recommendations for the evaluation criteria established in this study were followed, resulting in each group being divided into a group with appropriate FTD use and a group with potentially inappropriate FTD use: WS with appropriate FTD use (WS-A), WS with potentially inappropriate FTD use (WS-I), WM with appropriate FTD use (WM-A), WM with potentially inappropriate FTD use (WM-I), OS with appropriate FTD use (OS-A), OS with potentially inappropriate FTD use (OS-I), OM with appropriate FTD use (OM-A), and OM with potentially inappropriate FTD use (OM-I).

### The Evaluation Criteria to Identify Potentially Inappropriate FTD Use

We developed evaluation criteria to identify potentially inappropriate FTD use for noncancer pain, referring to the recommendations for safe use of prescription opioids by Korea Ministry of Food and Drug Safety and a literature review, which included the US CDC and Canadian guidelines [20,31-36]. Patients who received FTD once were evaluated against 2 criteria and those with multiple prescriptions were assessed using 5 criteria (Table 1). Compliance with all evaluation criteria indicated appropriate FTD use, whereas deviations from any criteria were indicative of potentially inappropriate FTD use.

The duration of FTD use per patch was considered to be 3 days, and cases where the period of each prescription was 90 days or less (ie, 30 patches or less) were evaluated as compliance with the recommendations. For patients with 2 or more prescriptions, even if the total consecutive use exceeded 90 days, it was deemed that the recommendations were followed as long as each prescription was within 90 days. To assess the dose for all types of opioids co-prescribed with FTD, we unified the doses of prescription opioids into MME. For FTDs, the MME per patch was calculated by multiplying the dose (mcg/h), the MME conversion factor (2.4), and the approved use period per patch (3 d). The MME/day was then calculated by dividing the MME by the longest prescription days among the co-prescribed opioids. For patients with multiple prescriptions, the estimated MME/day (eMME/day) was calculated considering the interval between visits. To evaluate the overlap in the prescription period covered by 2 consecutive prescriptions, patients were allowed to visit the health care institution up to 3 days earlier than scheduled. Flexibility in annual prescription periods was allowed based on national health insurance policies in South Korea. Overlapping prescriptions of up to 30 days within a 6-month period were permitted. Flexibility in prescription periods was granted with a 16% allowance for annual prescription periods, from the first visit to the last visit each year, of less than 182 days (6 mo), and a 30-day allowance for periods exceeding 182 days. Details of the evaluation method are provided in Multimedia Appendix 2: Methodology of evaluating FTD use using the established criteria.

**Table 1.** The established evaluation criteria for identifying potentially inappropriate use of fentanyl transdermal patch in patients with noncancer pain.

Criteria	Recommendations
For patients with a single FTD^a^ prescription per year	
Period	Prescribed for less than 90 days per prescription
Dose^b^	Prescribed at a dose of 90 MME^c^/day or less
For patients with multiple FTD prescriptions per year	
Period	Prescribed for less than 90 days per prescription
Dose^b^	Prescribed at a dose of 90 eMME^d^/day or less
Overlapping	Allowed up to 3 days of overlapping prescriptions for each prescription period Allowed up to 16% (maximum 30 days) of overlapping prescriptions for the annual prescription period
Multi-institutions	Prescribed by no more than 3 health care institutions in 1 year

a FTD: fentanyl transdermal patch.

b Dose was calculated by incorporating all types of opioids co-prescribed with FTD

c MME: morphine milligram equivalent.

d eMME:estimated morphine milligram equivalent.

### Statistical Analysis

Descriptive statistics were used to assess the clinical characteristics of the participants included in this study by group. The continuous variables, such as the number of reimbursements per patient, MME/day, annual FTD usage, and annual prescription opioid usage, were represented as means with SDs. We performed the Shapiro-Wilk test for normality and the Levene test for homogeneity of variance. The statistical methods used were ANOVA with the Tukey HSD for data satisfying both normality and homogeneity and the Kruskal-Wallis H test with the Dunn test for data violating normality. The categorical variables, including sex, age group, and diagnoses, were calculated as percentages or frequencies, and statistical significance was evaluated using *χ*^2^ test [37]. Additionally, a modified Lorenz curve was used to compare the working-age and older adult groups to evaluate the difference in FTD use [38]. In each group, modified Lorenz curves were drawn for FTD use from the top 1% to 100% of the 4 groups divided according to number of prescriptions and evaluation results. To determine the equality of FTD use across groups, we also plotted a classic Lorenz curve and calculated the Gini coefficient [39]. All data processing and statistical analysis were performed using R software (version 3.5.1; R Project for Statistical Computing).

### Ethical Considerations

This study followed the principles of the Declaration of Helsinki and was approved by the Ethics Committee of Hanyang University (HYUIRB-202307-008), and the informed consent was waived off by the review boards due to the nature of this research. All data in the manuscript and supplementary materials were anonymized in accordance with ethical standards, ensuring no personally identifiable information could be discerned.

## Results

### Trend in FTD Use

**Figure 1.**
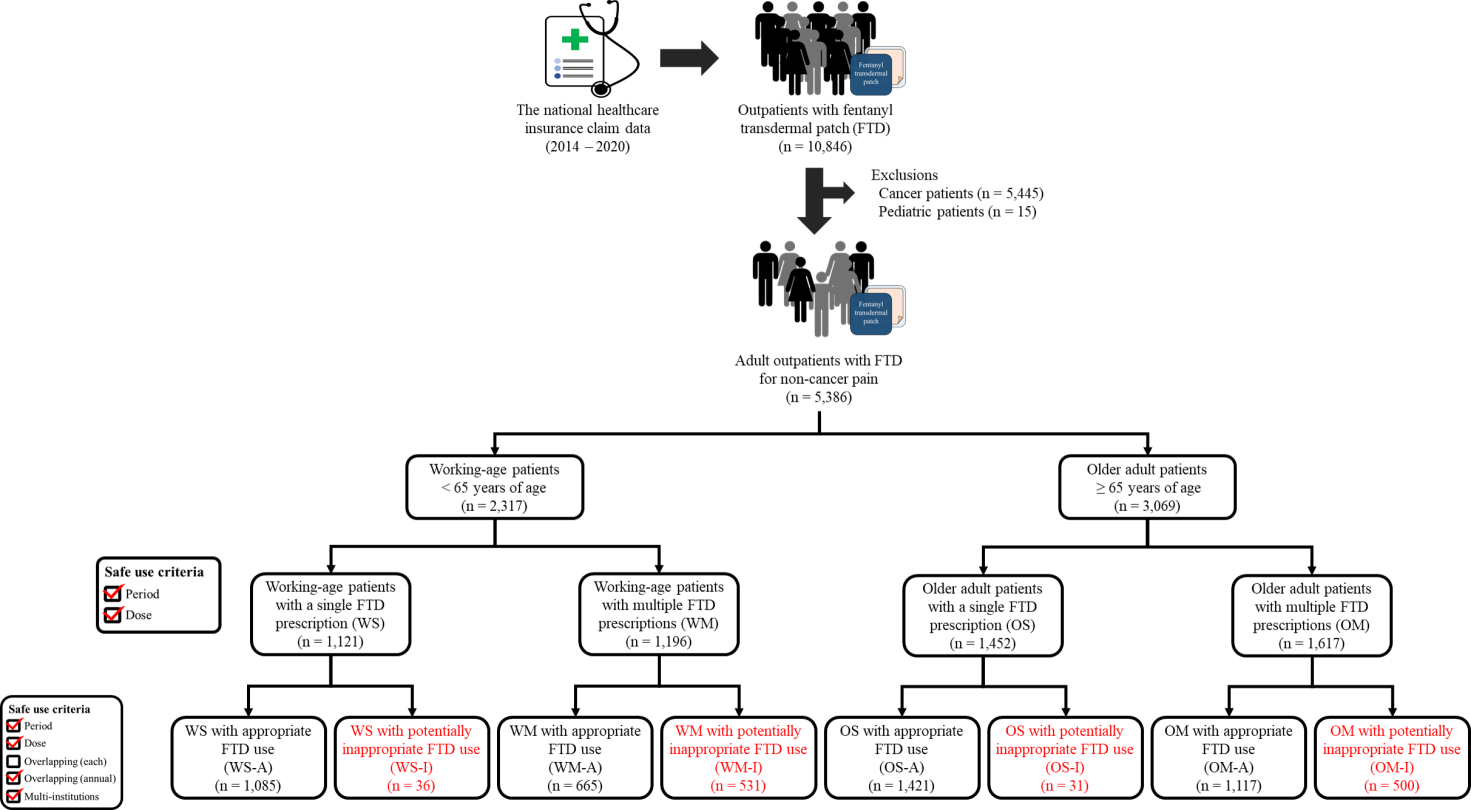
A schematic diagram of the patient classification process in the present nationwide cross-sectional study for evaluating fentanyl transdermal patch (FTD) use for noncancer pain in South Korea from 2014 to 2020. Patients were categorized into 8 groups by annual prescription frequency and compliance with evaluation criteria. OM: older adult patients with multiple FTD prescriptions; OM-A: older adult patients with multiple FTD prescriptions with appropriate FTD use; OM-I: older adult patients with multiple FTD prescriptions with potentially inappropriate FTD use; OS: older adult patients with a single FTD prescription; OS-A: older adult patients with a single FTD prescription with appropriate FTD use; OS-I: older adult patients with a single FTD prescription with potentially inappropriate FTD use; WM: working-age patients with multiple FTD prescriptions; WM-A: working-age patients with multiple FTD prescriptions with appropriate FTD use; WM-I: working-age patients with multiple FTD prescriptions with potentially inappropriate FTD use; WS: working-age patients with a single FTD prescription; WS-A: working-age patients with a single FTD prescription with appropriate FTD use; WS-I: working-age patients with a single FTD prescription with potentially inappropriate FTD use.

The insurance claim data of 9,284,003 registrants were evaluated, and 10,846 of these patients used FTDs in an outpatient setting. Following the exclusion of patients with diagnoses for cancer and pediatric patients, 5386 patients and their 19,800 reimbursements were included in the study. The study schematic diagram is shown in Figure 1.

The trends in the use of FTD are shown in Table 2. The number of patients with FTDs for noncancer pain increased from 58.6 per 100,000 registrants in 2014 to 61.3 per 100,000 registrants in 2018 (+4.6% over 4 years), and then decreased to 53.7 per 100,000 registrants in 2020 (–12.6% over 2 years). Meanwhile, the number of reimbursements increased by 7.4%, from 203.2 per 100,000 registrants in 2014 to 218.1 per 100,000 registrants in 2020, and by 1.4% in annual percent change (APC) terms. It indicated an increase in the number of FTD prescriptions per patient. In fact, the number of annual reimbursements per patient increased from 3.5 to 4.1 during the study period, and the average annual FTD usage per patient increased from 3216.7 MME to 4625.7 MME. Approximately 40% (2173/5401) of the study patients were male, which was less than females, but the male proportion steadily increased from 36.0% (21.1/58.6 per 100,000 registrants) in 2014 to 45.1% (24.2/53.7 per 100,000 registrants) in 2020. The proportion of older adult patients aged 65 years or older increased from 55.2% (32.3/58.6 per 100,000 registrants) in 2014 to 59.7% (32.1/53.7 per 100,000 registrants) in 2020.

**Table 2.** Annual trends in the use of fentanyl transdermal patches among patients with noncancer pain in South Korea, based on nationwide claim data from 2014 to 2020.

	Calendar year						
	2014	2015	2016	2017	2018	2019	2020
Number of patients, n per 100,000 registrants	58.6	57.4	56.9	61.0	61.3	54.6	53.7
Gender, n per 100,000 registrants (%)							
Male	21.1 (36.0)	21.8 (37.9)	22.8 (40.1)	25.2 (41.3)	25.4 (41.4)	22.6 (41.4)	24.2 (45.1)
Female	37.5 (64.0)	35.6 (62.1)	34.1 (59.9)	35.8 (58.7)	35.9 (58.6)	32.0 (58.6)	29.5 (54.9)
Age, n per 100,000 registrants (%)							
Working age	26.3 (44.8)	25.0 (43.5)	24.7 (43.4)	26.4 (43.2)	26.5 (43.3)	22.2 (40.7)	21.6 (40.3)
Older adult	32.3 (55.2)	32.4 (56.5)	32.2 (56.6)	34.6 (56.8)	34.8 (56.7)	32.4 (59.3)	32.1 (59.7)
Number of reimbursements, n per 100,000 registrants	203.2	201.8	213.3	222.5	219.6	216.9	218.1
Number of reimbursements, n per patient^a^	3.5 (5.1)	3.5 (4.1)	3.8 (4.8)	3.6 (4.8)	3.6 (5.2)	4.0 (5.3)	4.1 (6.1)
Average annual FTD usage, MME^b^	3216.7 (6063.2)	3664.4 (6564.4)	3840.9 (7240.9)	4480.4 (19,917.1)	4189.0 (10,968.7)	4361.9 (8281.8)	4625.7 (7478.1)

a The number of reimbursements per patient and annual FTD usage are presented as mean (SD). The percentages are calculated based on the number of patients and reimbursements calculated in each year.

b MME: morphine milligram equivalents.

### Patterns of FTD Use by Age Group

Patients were classified into working-age and older adult groups, comprising 2317 and 3069 patients (25.0 and 33.1 per 100,000 registrants), respectively. The male proportion was 50.6% (1172/2317) in working-age group, while 32.3% (990/3069) of older adult patients were male. There was a statistically significant difference between the 2 groups (*P*<.001). The working-age group had an average of 3.9 reimbursements per year, with an average MME/day of 64.4 for each reimbursement. The older adult group had an average of 3.5 reimbursements per year, and their average MME/day was 47.9, which was significantly lower than that of the working-age group (*P*=.005). The most common morbidity in both groups was diseases of the musculoskeletal system and connective tissue, accounting for 50.0% (4539/9075) in the working-age group and 61.0% (6537/10,725) in the older adult group. In the working-age group, the FTD prescriptions for the diseases of the circulatory system were less common (2.4% vs 5.5%), but for the diseases of the nervous system and diseases of the digestive system, the FTD prescriptions were more frequent compared to the older adult group (8.6% vs 6.8%; 4.2% vs 1.6%, respectively).

Dividing the groups by the number of FTD prescriptions, the WM had an average of 6.7 reimbursements, and the average annual total amount of FTDs used was approximately 18.7 times greater than the total for the WS (8938.9 vs. 477.5 MME). The OM showed a smaller difference: an average of 5.7 reimbursements and 10.2 times more FTD total usage than OS. Calculating MME/day for each prescription, WS, WM, OS, and OM were prescribed an average dose of 39.7, 67.9, 38.3, and 49.4 MME/day, respectively (Table 3).

**Table 3.** The clinical characteristics of 4 groups stratified by age and prescription frequency among patients with noncancer pain prescribed fentanyl transdermal patch in South Korea from 2014 to 2020.

	Working-age group			Older adult group			*P* value^a^
	WS	WM	Total	OS	OM	Total	
Number of patients, n	1121	1196	2317	1452	1617	3069	-
Gender, n (%)^b^							<.001
Male	539 (48.1)	633 (52.9)	1172 (50.6)	418 (28.8)	572 (35.4)	990 (32.3)	
Female	582 (51.9)	563 (47.1)	1145 (49.4)	1034 (71.2)	1045 (64.6)	2079 (67.7)	
Number of reimbursements, n	1121	7954	9075	1452	9273	10,725	-
Number of reimbursements, n per patient^c^	1.0 (0.0)	6.7 (6.8)	3.9 (5.7)	1.0 (0.0)	5.7 (5.2)	3.5 (4.4)	.005
Annual usage, MME^d^							
FTD	477 (564)	8939 (20,247)	4845 (15,151)	580 (562)	5890 (7320)	3378 (5950)	<.001
Prescription opioids	555 (824)	12,043 (24,828)	6485 (18,744)	651 (846)	7380 (14,401)	4196 (10,994)	<.001
Prescription opioid usage per reimbursement, MME/day	39.7 (24.9)	67.9 (53.5)	64.4 (51.7)	38.3 (23.7)	49.4 (37.2)	47.9 (35.9)	<.001
Primary diagnosis, n (%)^b^							<.001
Diseases of the musculoskeletal system and connective tissue	672 (59.9)	3867 (48.6)	4539 (50.0)	859 (59.2)	5678 (61.2)	6537 (61.0)	
Injury, poisoning and certain other consequences of external causes	201 (17.9)	719 (9.0)	920 (10.1)	280 (19.3)	833 (9.0)	1113 (10.4)	
Diseases of the nervous system	35 (3.1)	748 (9.4)	783 (8.6)	45 (3.1)	687 (7.4)	732 (6.8)	
Diseases of the digestive system	30 (2.7)	352 (4.4)	382 (4.2)	24 (1.7)	148 (1.6)	172 (1.6)	
Diseases of the circulatory system	23 (2.1)	194 (2.4)	217 (2.4)	59 (4.1)	530 (5.7)	589 (5.5)	
Others	160 (14.3)	2074 (26.2)	2234 (24.7)	185 (12.6)	1397 (15.1)	1582 (14.7)	

a *P* value means the comparison between the total working-age and older adult groups.

b The percentages are calculated based on the number of patients and reimbursements calculated in each group.

c The number of reimbursements per patient, annual usage and prescription opioid usage per reimbursement are presented as mean (SD)

d MME: morphine milligram equivalent

### Identification of Potentially Inappropriate FTD Use

Potentially inappropriate FTD use was assessed in 24.5% (567/2317) of working-age patients and 17.3% (531/3069) older adult patients. Among them, 67 were prescribed FTD once (WS-I, 36; OS-I, 31) and 1031 used FTDs more than twice (WM-I, 531; OM-I, 500). The proportion of male patients in WM-I was 56.7% (301/531), meaning that while 47.6% (301/633) of male patients in the WM were evaluated as having potentially inappropriate FTD use, only 40.9% (230/563) of females were potentially inappropriate FTD users. In older adult patients with multiple FTD prescriptions, 36.5% (209/572) and 27.8% (291/1045) of males and females, respectively, were assessed as potentially inappropriate FTD users, which was lower than that of the WM.

The proportions of patients identified as potentially inappropriate FTD users for each criterion. For 2 single-prescription groups, high doses of prescriptions were the main reason for being evaluated as potentially inappropriate: 3.1% (35/1121) of WS and 2.1% (30/1421) of OS. The primary reason for potentially inappropriate evaluation in the 2 multiple-prescription groups was overlapping of each prescription (33.6% of WM; 402/1196 and 25.8% of OM; 417/1617), followed by high-dose prescriptions and overlapping of annual prescriptions (17.8% of WM; 213/1196 and 9.8% of OM; 159/1617). Regarding evaluation criterion for dose, the proportion showed the greatest difference at 24.2% (290/1196) for working-age patients and 11.7% (189/1617) for older adult patients. Patients with multiple health care institution visits and those who received long-term treatment were both less than 1% in each group.

**Figure 2.**
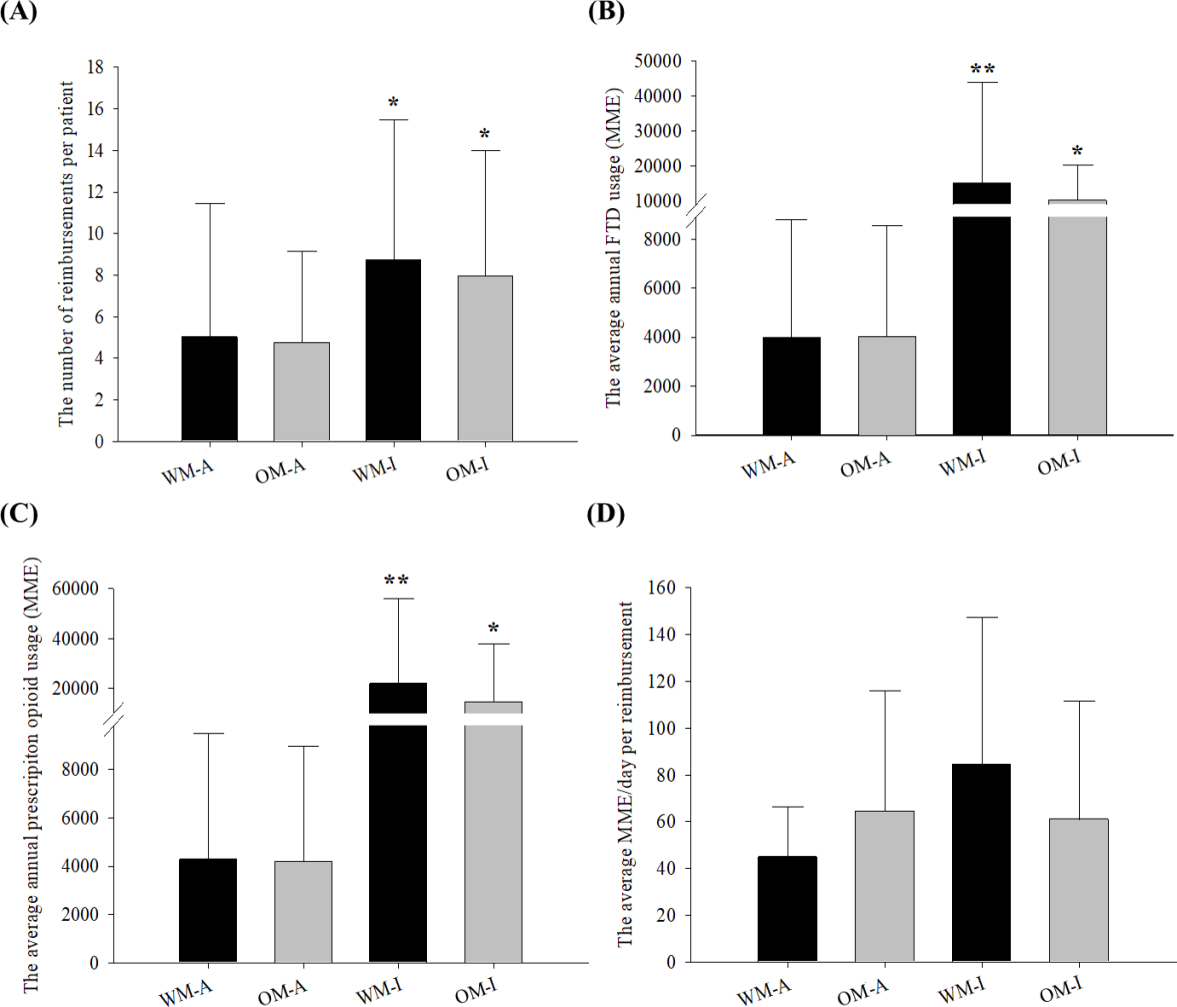
Comparison of the characteristics of 4 groups with multiple fentanyl transdermal patch prescriptions, stratified by age and evaluation outcome, in a nationwide cross-sectional study of patients with noncancer pain in South Korea using claim data from 2014 to 2020. Panel A shows the number of reimbursements per patient; Panel B shows the average annual FTD usage; Panel C shows the average annual prescription opioid usage; and Panel D shows the average MME/day per reimbursement. Black bars represent working-age patients and the older adult patients are represented by gray bars. *Means *P*<.001 compared to the corresponding group with appropriate FTD use. **Means *P*<*.*001 compared to the other groups. MME: morphine milligram equivalent; OM-A: older adult patients with multiple FTD prescriptions with appropriate FTD use; OM-I: older adult patients with multiple FTD prescriptions with potentially inappropriate FTD use; WM-A: working-age patients with multiple FTD prescriptions with appropriate FTD use; WM-I: working-age patients with multiple FTD prescriptions with potentially inappropriate FTD use.

Figure 2 presents a comparative analysis of characteristics related to FTD and prescription opioid usage across 4 multiple-prescription groups. As shown in **Figure 2A**, both the WM-I and OM-I groups had significantly more numerous reimbursements per patient than the corresponding group with appropriate FTD use (WM-I, 8.7 vs WM-A, 5.0; OM-I, 7.9 vs OM-A 4.8) but there were no significant differences between the WM-I and OM-I. Similar trends were observed for the average annual FTD usage and prescription opioid usage (****Figure** 2B and 2C**). The WM-I had a significantly higher average annual FTD usage (3.8 times; *P*<0.001) and prescription opioid usage (5.1 times; *P*<.001) than those of the WM-A. The OM-I also had significantly higher average annual FTD use and prescription opioid use compared to the OM-A (*P*<.001). In addition to the comparison with potentially inappropriate users, the WM-I had significantly higher average annual FTD and prescription opioid usage than the OM-I (*P*<.001). For the average MME/day per reimbursement, the WM-I showed significantly greater MME/day than the WM-A, while the MME/day of OM-I was lower than that of OM-A, although there was no significant difference (**Figure 2D**). Comparing the eMME/day for multiple-prescription groups to MME/day, the appropriate groups showed a lower eMME/day than their average MME/day (WM-A, 32.7 eMME/day vs 44.9 MME/day; OM-A, 29.3 eMME/day vs 64.4 MME/day), while potentially inappropriate groups showed a higher value of eMME/day than their MME/day (WM-I, 149.4 eMME/day vs 84.4 MME/day; OM-I, 85.6 eMME/day vs 60.9 MME/day). Among inappropriate groups, WM-I had significantly higher eMME/day than OM-I (*P*<.001).

### Inequality Assessment of FTD Use

**Figure 3.**
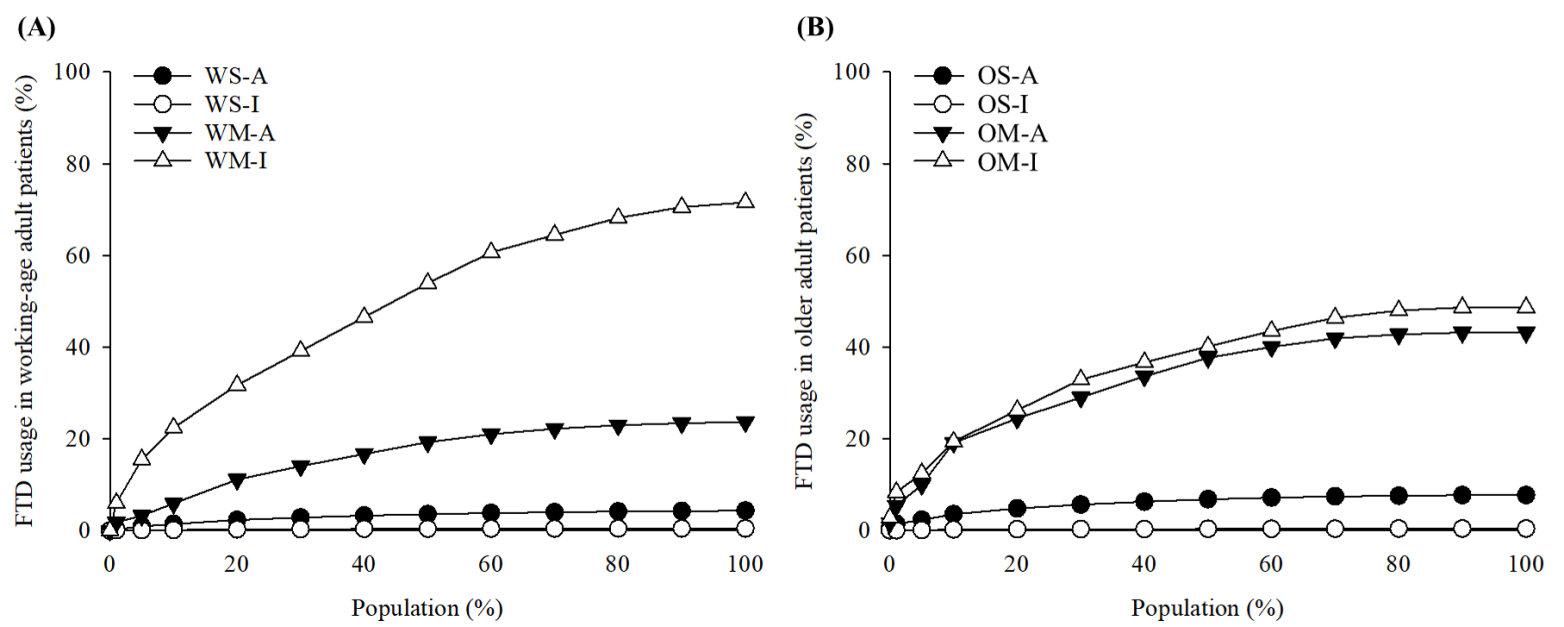
A modified Lorenz curve illustrating the distribution of fentanyl transdermal patch (FTD) usage among patients with noncancer pain in South Korea from 2014 to 2020, stratified by evaluation outcome and prescription frequency. Panel A presents data for the working-age group, and Panel B presents data for older adult group. OM-A: older adult patients with multiple FTD prescriptions with appropriate FTD use; OM-I: older adult patients with multiple FTD prescriptions with potentially inappropriate FTD use; OS-A: older adult patients with a single FTD prescription with appropriate FTD use; OS-I: older adult patients with a single FTD prescription with potentially inappropriate FTD use; WM-A: working-age patients with multiple FTD prescriptions with appropriate FTD use; WM-I: working-age patients with multiple FTD prescriptions with potentially inappropriate FTD use; WS-A: working-age patients with a single FTD prescription with appropriate FTD use; WS-I: working-age patients with a single FTD prescription with potentially inappropriate FTD use.

The total amount of FTDs used in working-age patients and older adult patients was 11,226,138 MME and 10,365,939 MME, respectively. To confirm the distribution of FTD usage for each group, a modified Lorenz curve was plotted against the total amount of FTDs (Figure 3). The WM-I accounted for more than 70% (8,034,172/11,226,138) of the total FTDs used in working-age patients. In particular, the amount of FTDs used by the top 5% of the WM-I was 3.6 times the amount of FTDs used by patients in the top 5% of the other 3 groups. With only 36 patients in the WS-I, the use in this group was only 0.44% of that in the working-age patients. In the older adult patients, the OM-I also used the highest proportion of FTDs, but unlike the working-age group, there was only a small difference between the OM-I and OM-A (48.7%; 5,045,751/10,365,939 vs 43.2%; 4,477,804/10,365,939). The OS-I also had a very low portion of 0.41%, as there were only 31 patients in the OS-I. Moreover, Gini coefficients were calculated for the classic Lorenz curves of all groups. The working-age patients had a greater Gini coefficient than the older adult group, indicating unequal use of FTDs (0.461 vs 0.406). Each of the working-age groups showed greater inequality than the corresponding older adult groups.

## Discussion

### Principal Findings and Comparison With Previous Works

This study is the first study to assess the rational use of FTDs with recommendations for safe use of prescription opioids using a nationwide database. This study identified 567 (24.5%) working-age patients and 531 (17.3%) older adult patients as potentially inappropriate FTD users over a 7-year period.

The number of FTD users for noncancer pain in South Korea exhibited an increase from 2014 to 2018, followed by a subsequent decline from 2019, resulting in an overall reduction of 8.4% during the study period. The decline in the number of patients prescribed FTDs in 2018 coincides with the implementation of the NIMS in South Korea. The NIMS is a system designed to monitor and report on the manufacturing, distribution, prescribing, and dispensing of all controlled pharmaceutical drugs in the country [15]. It is intended to serve some of the functions of the prescription drug monitoring program in the United States, potentially contributing to the decrease in unnecessary use of FTDs. However, the number of FTD prescriptions per 1000 registrants increased over the study period. The increase rate found in this study is consistent with the APC of 1.8% for all types of opioid prescriptions in South Korea from 2013 to 2019 [7]. The trend in the number of FTD prescriptions per patient, rather than per population, is more rapidly increasing, with an APC of 2.6%. The average annual amount of FTD usage per patient increased at an APC of 6.9%, suggesting that the increase in FTD usage amount is driven not only by an increase in the number of FTD prescriptions per patient but also by an increase in the FTD usage per prescription. These changes in the number of patients and FTD prescribing trends were also supported by the results that the decrease in the number of patients with a single FTD prescription and the increase in the number of patients with multiple FTD prescriptions. Additionally, the increase in FTD usage (total, 13.6 times; working age, 18.7 times; older adult, 10.2 times) surpassed the increase in the average number of reimbursements per patient (total, 6.1 times; working age, 6.7 times; older adult, 5.7 times) between the 2 groups. According to a previous study, prescription opioid usage in South Korea increased at an APC of 30.3% from 2015 to 2019, while in the United States and Canada, it declined during the same period by 14.1% and 11.6% per year, respectively [12]. Another study reported that the amount of fentanyl and its derivatives used per patient in the United States decreased by 65.5% from 2010 to 2019 [40]. Despite the lower population-based prescription opioid usage in South Korea compared to global levels in 2019 [13], approximately 20% of patients using FTDs for noncancer pain were deemed at risk for potentially inappropriate use of prescription opioids, including FTDs.

We developed evaluation criteria based on practice guidelines from different countries and endeavored to reflect what clinicians might encounter in the real world. For instance, the prescription period was assessed only in terms of the number of days covered by each prescription, regardless of the duration of consecutive use. It is based on the assumption that clinicians evaluate the necessity of FTD use for each patient during the re-prescription process. Overlapping prescriptions were evaluated by setting a margin of flexibility, allowing 3 days per prescription and 16% per year, considering the practical problems in the real world. For patients with multiple FTD prescriptions, eMME/day was calculated and used. The MME/day is the patient’s prescribed opioid dose divided by the estimated treatment duration based on the authorized dosage, while the eMME/day is the prescription opioid dose divided by the time until the next visit, assuming the patient has taken all of the prescribed opioids prior to the visit. If the patient is compliant with a prescribed regimen, the MME/day and eMME/day should be equal. The eMME/day may be higher than MME/day for patients who visit earlier than scheduled, and eMME/day may be lower than MME/day for those who visit later than scheduled.

This study showed that a higher proportion of working-age patients was identified as potentially inappropriate FTD users than older adult patients (24.5% vs 17.3%). Comparing the 2 groups with the multiple FTD prescriptions, 44.4% of the working-age patients were evaluated as having potentially inappropriate FTD use, while only 30.9% of the older adult patients were classified into the OM-I. Although the numbers of patients in the WM-I and OM-I were similar, the total annual amount of FTD use was 1.6 times higher in the WM-I than in the OM-I. In addition, there were more patients evaluated in WM-I than in OM-I as potentially inappropriate FTD users due to high doses. Compared to the corresponding groups with appropriate FTD use, the WM-I used 3.0 times that of the WM-A patients, while the OM-I used only 1.1 times that of the OM-A. These findings suggest that the WM-I patients frequently used FTDs outside practice guidelines more than the OM-I patients. The comparison of Gini coefficients calculated by plotting the classic Lorenz curve also revealed that the FTD use was relatively more equally distributed in the older adult patients than in the working-age patients. Additionally, in both WM-A and OM-A, MME/day was higher than the newly calculated eMME/day, but in both WM-I and OM-I, eMME/day was higher than MME/day. This suggests that patients who used FTD appropriately may use them longer than prescribed periods, while the patients having a risk of potentially inappropriate FTD use may take a higher than prescribed dose of FTDs or hoard spare FTDs. The difference from MME/day to eMME/day was greater in the WM-I than in the OM-I. There was a 1.4 times larger increase in OM-I patients (mean difference of 24.7), but a 1.8 times larger increase in WM-I patients (mean difference 64.9). These differences also support a more potentially inappropriate FTD use in working-age patients. These findings are consistent with a result of meta-analysis finding that the risk of opioid overdose was greater in adults aged 25‐54 than in the older adult [28]. Furthermore, death rates from prescription opioid overdoses were also reported to be higher in younger patients than in older patients [41]. Prescription opioid-related death in young adults might have a significant impact in terms of years of life lost, increasing the public health burden [42].

The strength of this study is that we focused on the potentially inappropriate FTD use, which has recently been assessed as having the highest risk of misuse among prescription opioids. Moreover, this study used national claim data to investigate changes in FTD use for noncancer pain and assessments of potentially inappropriate FTD use simultaneously, which resulted in more consistent results.

### Limitations

This study has several limitations. First, it was a cross-sectional analysis based on health care insurance claims data, which means it only included data for which FTD claims were submitted by health care institutions. Consequently, data regarding nonclaimed use of FTDs were not captured in this study. Second, despite several adjustments having been made to the evaluation criteria, there remains a potential risk that patients who are deemed by clinicians to require high doses or long-term use of FTDs for pain management may be classified as inappropriate users. Third, the FTD usage was assessed solely based on prescriptions, without verification of actual patient consumption. This limitation suggests that patient adherence may have influenced our results.

### Conclusions

In South Korea, although the number of patients receiving FTD prescriptions has decreased, the total amount of FTDs consumed has increased in recent years, suggesting that caution is warranted. The present study demonstrated the potential for inappropriate FTD use in working-age patients compared with older adult patients. While our findings don’t imply that FTDs should be restricted to those patients who desperately need them, health care providers should be alert to the risk of prescription opioids and the opioid epidemic and promote the appropriate use of prescription opioids. Further research is needed to quantify and qualify the risk factors in patients having a risk of potentially inappropriate use, considering the clinical rationale associated with prescribing FTDs.

## Supplementary material

10.2196/63960 Multimedia Appendix 1 Strengthening the Reporting of Observational Studies in Epidemiology (STROBE) Checklist.

10.2196/63960 Multimedia Appendix 2 Methodology of evaluating FTD use using the established criteria: (A), calculation of MME/day for FTDs; (B), MME/day calculation for co-prescribed opioids; (C), eMME/day calculation in a potentially inappropriate FTD use case; (D), eMME/day calculation in an appropriate FTD use case; (E), overlapping evaluation (annual).

## Abbreviations

APC: annual percent change
CDC: Centers for Disease Control and Prevention
eMME: estimated morphine milligram equivalents
FTD: fentanyl transdermal patch
HIRA: Health Insurance Review and Assessment Service
MME: morphine milligram equivalent per day
NIMS: Narcotics Information Management System
NPS: National Patient Sample
OM: older adult patients with multiple FTD prescriptions
OM-A: older adult patients with multiple FTD prescriptions with appropriate FTD use
OM-I: older adult patients with multiple FTD prescriptions with potentially inappropriate FTD use
OS: older adult patients with a single FTD prescription
OS-A: older adult patients with a single FTD prescription with appropriate FTD use
OS-I: older adult patients with a single FTD prescription with potentially inappropriate FTD use
OUD: opioid use disorder
PDMP: prescription drug monitoring program
STROBE: Strengthening the Reporting of Observational Studies in Epidemiology
WM: working-age patients with multiple FTD prescriptions
WM-A: working-age patients with multiple FTD prescriptions with appropriate FTD use
WM-I: working-age patients with multiple FTD prescriptions with potentially inappropriate FTD use
WS: working-age patients with a single FTD prescription
WS-A: working-age patients with a single FTD prescription with appropriate FTD use
WS-I: working-age patients with a single FTD prescription with potentially inappropriate FTD use
