# Supplementary figures and images for: Potentially Inappropriate Use of Transdermal Fentanyl in Working-Age and Older Adult Populations with Non-Cancer Pain: Nationwide Cross-Sectional Study
Source: JMIR Public Health Surveill. 2025 May 28;11:e63960. doi: 10.2196/63960 (PMC12165270; doi:10.2196/63960)

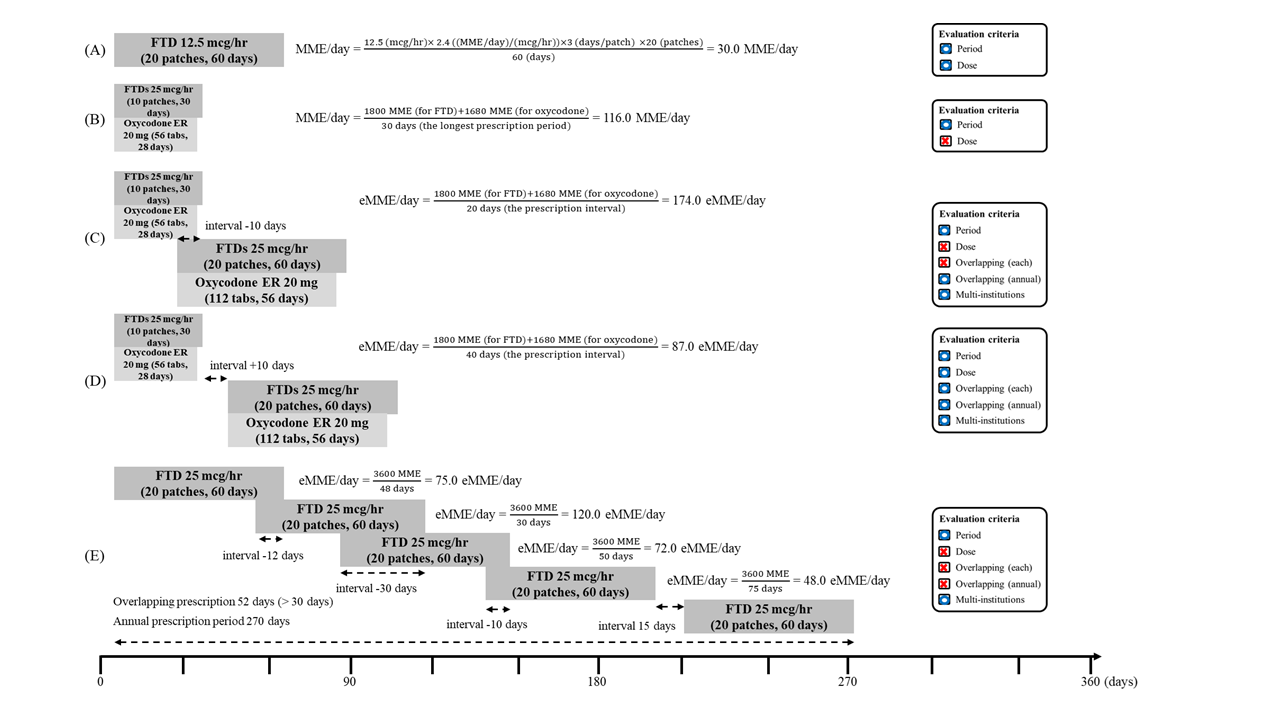

Supplement: Multimedia Appendix 2 [file publichealth-v11-e63960-s002.png]
